# Atrial fibrillation in cancer survivors – a systematic review and meta-analysis

**DOI:** 10.1186/s40959-023-00180-3

**Published:** 2023-06-17

**Authors:** Yueyang Bao, John Lee, Udit Thakur, Satish Ramkumar, Thomas H. Marwick

**Affiliations:** 1 School of Medicine, University of Tasmania, Hobart, TAS Australia; 2 Department of Cardiology, Royal Hobart Hospital, Hobart, TAS Australia; 3 Monash Cardiovascular Research Centre and MonashHeart, Monash Health, Melbourne, VIC Australia; 4 Baker Heart and Diabetes Institute, 75 Commercial Road, Melbourne, VIC 3004 Australia

**Keywords:** Atrial fibrillation, Screening, Survivorship

## Abstract

**Background:**

Atrial fibrillation (AF) is a common cardiac complication during cancer treatment. It is unclear if cancer survivors have increased AF risk when compared to the population. AF screening is now recommended in patients ≥65 years, however there are no specific recommendations in the oncology population. We sought to compare the AF detection rate of cancer survivors compared to the general population.

**Methods:**

We searched the Pubmed, Embase and Web of Science databases using search terms related to AF and cancer mapped to subject headings. We included English language studies, limited to adults > 18 years who were > 12 months post completion of cancer treatment. Using a random-effects model we calculated the overall AF detection rate. Meta-regression analysis was performed to assess for potential causes for study heterogeneity.

**Results:**

Sixteen studies were included in the study. The combined AF detection rate amongst all the studies was 4.7% (95% C.I 4.0-5.4%), which equated to a combined annualised AF rate of 0.7% (95% C.I 0.1–0.98%). There was significant heterogeneity between studies (*I*^*2*^ = 99.8%, p < 0.001). In the breast cancer cohort (n = 6 studies), the combined annualised AF rate was 0.9% (95% C.I 0.1–2.3%), with significant heterogeneity (*I*^*2*^ = 99.9%, p < 0.001).

**Conclusion:**

Whilst the results should be interpreted with caution due to study heterogeneity, AF rates in patients with cancer survival >12 months were not significantly increased compared to the general population.

**Study Registration:**

Open Science Framework - DOI: 10.17605/OSF.IO/APSYG.

**Supplementary Information:**

The online version contains supplementary material available at 10.1186/s40959-023-00180-3.

Atrial fibrillation (AF) is often diagnosed in patients during cancer treatment, provoked by electrolyte abnormalities, cardiotoxicity, infection and systemic inflammation [1, 2]. The prevalence of AF in the general population (1–2%) is exceeded in cancer patients [1, 3], driven by common risk factors such as obesity and diabetes mellitus, as well as age [4, 5]. A recent prospective study reported that 19% of patients with cancer developed AF during a mean follow-up time of 16.3 years, compared with 9% of patients without cancer, with cancer itself being an independent risk factor for AF [6, 7]. Along with traditional AF risk factors, the contributing factors for increased AF risk in cancer survivors may include cardiotoxicity related to cancer therapy as well as a pro-inflammatory state. There are oncological-specific factors, with certain cancer types (e.g. breast cancer) and those with more advanced cancer stages, being associated with higher AF risk [8, 9]. Finally, cancer therapies themselves - such as radiotherapy, taxanes, tyrosine kinase inhibitors and proteasome inhibitors - can promote AF. The mechanism may involve a pro-inflammatory state mediated by increased production of cytokines and chemokines potentially leading to atrial remodelling [7].

The development of AF in cancer survivors has important clinical implications. As well as the potential for debilitating symptoms, AF may exacerbate pre-existing cancer therapy-related cardiotoxicity. AF may also be associated with an increased thromboembolic risk, which is potentially higher than non-cancer patients with non-valvular AF. Traditional AF risk scores such as CHA_2_DS_2_-VASC may underestimate stroke risk in cancer survivors as they do not incorporate other cancer specific risk factors [10]. Nonetheless, recent clinical guidelines advocating routine AF screening in patients ≥65 years [11] have been controversial, and there are no specific recommendations in recently published cardio-oncology guidelines [12]. AF screening and use of anticoagulation therapy in this cohort of patients may be associated with improved health outcomes and reduction in cardiovascular complications. The aim of this systematic review was to investigate the rates of AF in cancer survivors and to compare this to the general population.

## Materials and methods

This study was conducted in accordance with the Preferred Reporting Items for Systematic Reviews and Meta-Analyses (PRISMA) guidelines [13].

*Search Strategy.* A comprehensive search of the PubMed, Embase, and Web of Science electronic databases was performed to include articles from the databases’ date of inception to October 31st, 2022. Key terms included “cancer,” “neoplasm,” “carcinoma,” “malignancy,” “tumour,” “atrial fibrillation,” “atrium,” “cardiovascular events,” and “cardiac outcomes” which were mapped to subject headings. Our search was limited to adult human subjects > 18 years and limited to the English language. The specific search strategies for each database are available in the published protocol.

***Study Selection.*** Two investigators (Y.B. and J.L.) independently screened titles and abstracts of retrieved articles. Studies which investigated rates of AF in cancer patients were selected for full-text screening for eligibility if they included patients in cancer remission > 18 years old. We confined the study to patients who were > 12 months post completion of cancer treatment (either chemotherapy, radiotherapy, or immunotherapy), in order to avoid including AF outcomes during cancer treatment where there may be other confounders such as sepsis or electrolyte abnormalities. The primary outcome measure was the annualized rate of AF. Articles were excluded if cancer patients were < 18 years old, < 12 months from cancer treatment, or if patients only underwent radical surgical treatment. We also excluded non-English articles, case studies or editorials. Disagreements between investigators were resolved through consensus or consultation with a third investigator (S.R.).

***Data Extraction.*** Two investigators (Y.B. and J.L.) independently performed data extraction using a standardized data extraction form, including study characteristics, patient demographics, co-morbidities, cancer types and treatments, length of follow-up, AF screening and definition and AF detection rate. If the data was not readily available in the manuscript or were unclear, we contacted the study authors for clarification and/or additional data.

***Statistical Analysis.*** AF detection rates of individual studies were converted to annualized risks. The cumulative annualized AF detection rate and 95% confidence interval was calculated using a random effects model. The results were displayed as a forest plot and heterogeneity amongst the studies was assessed using the *I*^2^ statistic. A subgroup analysis was performed investigating the annualised AF detection rate of breast cancer patients. Meta-regression analysis was performed to determine sources of study heterogeneity.

Risk of bias was independently assessed by two investigators (Y.B. and J.L.) and differences were resolved by discussion with a third author (S.R.). The risk of bias of cohort and case-control studies were assessed using the Newcastle-Ottawa Scale (NOS) [14], while a modified NOS was used for cross-sectional studies. We defined studies with NOS scores > = 7 stars as high quality and NOS score < 7 as low quality. Statistical analysis was performed using Stata v.13 (StataCorp, College Station, TX). The certainty of the evidence was evaluated using the GRADE guidelines [15].

## Results

**Fig. 1:**
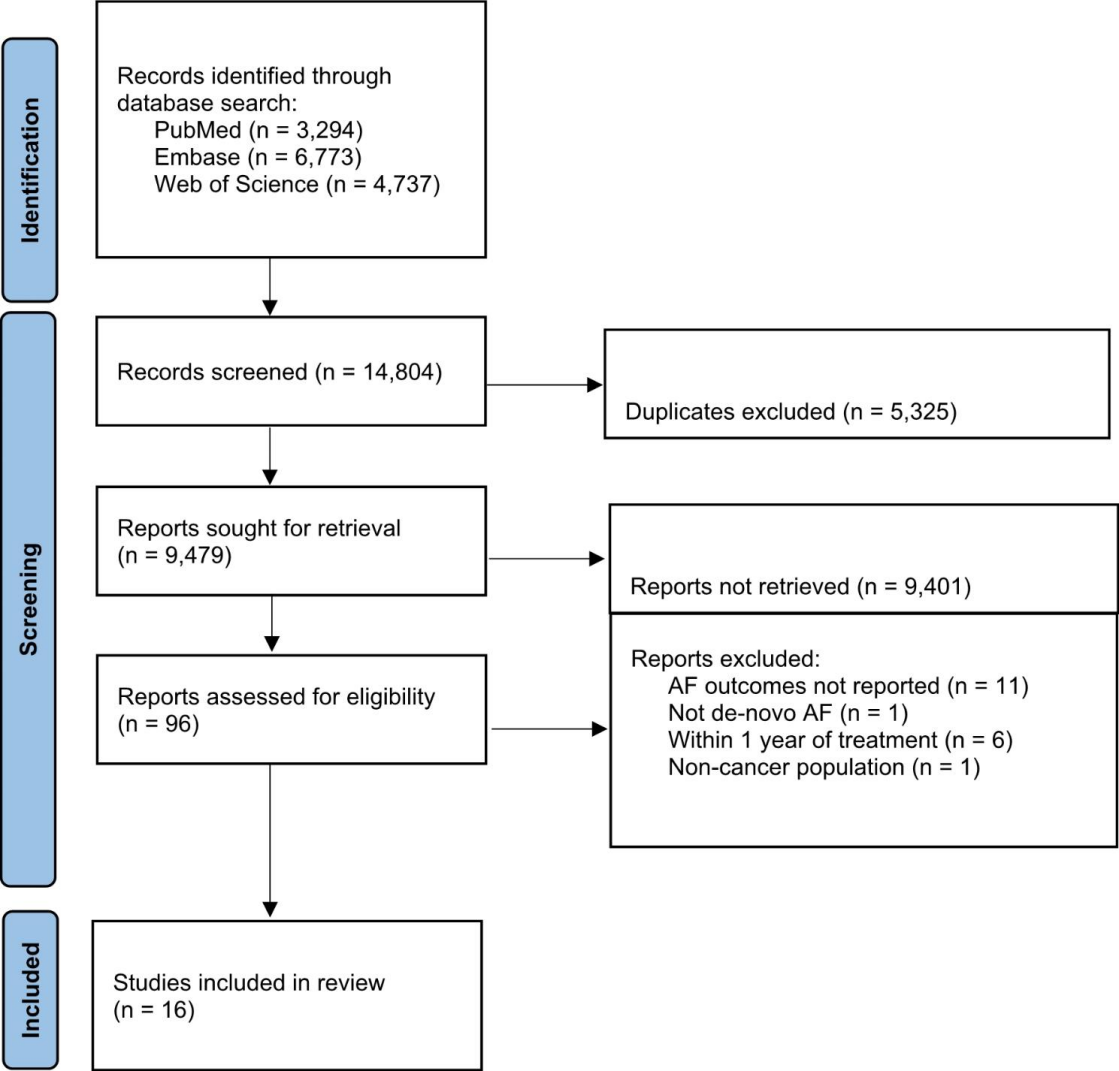
PRISMA flow diagram of the study selection

***Study characteristics.*** The initial search strategy across the three databases yielded 14,804 papers (Fig. 1). After removing duplicate articles, a total of 9479 articles’ titles and abstracts were screened for full-text eligibility. From these, 78 articles moved onto full-text screening, and 16 studies (comprising 1,406,464 cancer patients) were included [8, 16–29].

Of the 16 studies (Table 1), eleven were retrospective cohorts, one was prospective cohort, one was case-control, and three were cross-sectional. Three studies reported outcomes in esophageal cancer cohorts, one in thyroid cancer, six in breast cancer, one in non-Hodgkin lymphoma, one in endometrial, and four in various cancers combined. There was a significant range in age and gender. Vascular co-morbidities were reported in 13/16 studies, demonstrating significant variation amongst studies with high rates of hypertension. AF definitions were only provided in 6/16 studies and reporting of the methods used for AF detection was poor with the majority based on review of medical records or clinical diagnosed using 12 lead electrocardiograms. There were no studies documenting use of modern screening technologies such as smartwatches, monitoring patches or single lead ECG monitoring devices.

**Table 1:** Summary of included studies in systematic review

Study	Study Design	Cancer Type	Staging	Age (SD)	Cancer Patients (n)	Treatment	Median Follow-up (years ± SD)	AF Definition	AF Screening Method	Male (%)	HTN (%)	DM (%)	IHD (%)	CCF (%)	Previous stroke (%)	AF Rate (%)
Rao et al. (2012)(25)	Retrospective cohort	Oesophageal	NA	68	997	Chemotherapy (5-FU, CP, capecitabine, epirubicin), Oesophagectomy	NA	NA	12-lead ECG	71.1	23.7	7.9	11.1	NA	NA	21
Hesselink et al. (2015)(23)	Retrospective cohort	Thyroid	82.4% Tx – T3 17.6% T4 65.8% Nx-N0 34.2% N1 90.7% Mx-M0 9.3% M1	48.6 (14)	518	Thyroidectomy, Radioiodine Ablation, Thyroid Hormone Suppression Therapy	8.7	NA	ECG or diagnosed from cardiologist	25.3	17	4.2	NA	0.2	NA	6.8
O’Neal et al. (2015)(24)	Cross-sectional	Various	NA	70 (8.6)	2,248	NA	NA	NA	Scheduled ECG or self-reported history of physician diagnosis	59	55	20	NA	NA	NA	11
Chalazan et al. (2016)(29)	Retrospective cohort	Breast	NA	58.9 (12.8)	2,124	Chemotherapy (tyrosine kinase inhibitors, alkylating agents, monoclonal antibodies, antimetabolites, mitotic inhibitors, hormone modifiers, topoisomerase inhibitors, antineoplastic antibiotics), Radiotherapy	NA	Validated algorithm	NA	NA	NA	NA	NA	NA	NA	4
Sorigue et al. (2018)(27)	Retrospective cohort	Non-Hodgkin lymphoma	34% I – II 66% III – IV	61	724	Chemotherapy	5.3	NA	12-lead ECG	52	32	16	NA	NA	NA	5.5
Abdel-Qadir et al. (2019) (16)	Population-based, retrospective, matched cohort study	Breast	44.3% I 38.7% II 13.4% III 3.6% Unknown	60 (13)	68,113	Chemotherapy (anthracycline, trastuzumab, other), Radiotherapy, Mastectomy	5.7 (2.9)	Validated algorithm	NA	0	43.4	15.9	5.5	2.1	0.4	4.6
D’Souza et al. (2019)(18)	Retrospective cohort	Breast	NA	62	74,155	NA	3	Hospitalization with discharged diagnosis of AF	NA	0	19.1	4.4	3.7	1.2	NA	1.3
Jacobs et al. (2019)(28)	Retrospective cohort	Breast	NA	57.6 (13.4)	1,338	Radiotherapy	10	NA	ECG	0.9	NA	NA	NA	NA	NA	5.2
Hayashi et al. (2019)(19)	Retrospective cohort	Oesophageal	cT1N0M0 stage IA	66.5	80	Chemotherapy (5-FU, CP, nedaplatin), Radiotherapy	6.1	NA	NA	88.8	45	12.5	NA	NA	NA	1.25
Jakobsen et al. (2019)(20)	Retrospective registry-based cohort	Various	NA	67 (13.3)	316,040	NA	12	ICD-10 codes	NA	48.5	35.2	NA	5.5	2	6.2	5.7
Mery et al. (2020)(21)	Retrospective cohort	Breast	0.70% O 47.9% I 33.1% II 11.0% III 2.20% IV	62.5	682	Chemotherapy (5-FU, epirubicin, cyclophosphamide, docetaxel, DXR, PTX, CB), Radiotherapy, Surgery, Endocrine Therapy, Trastuzumab	5	NA	NA	0.9	73.8	17.4	NA	NA	NA	0.7
Li et al. (2021)(26)	Cross-sectional	Breast, Lung, Colorectal, Gastric, Thyroid	NA	69.12 (9.89)	35,861	NA	NA	NA	12-lead ECG or in 24-hour single-lead ECG recording or Holter	51.6	21.9	29.2	6.5	1	NA	2.9
Parahuleva et al. (2021)(22)	Prospective case-control	Endometrial	NA	63.34 (7.03)	310	Chemotherapy (PTX, CB, DXR, liposomal DXR, CP, docetaxel), Hormone Therapy, Surgery	2.5 (0.5)	NA	12-lead ECG, hospital discharge diagnosis, death certificate	0	NA	NA	NA	NA	NA	11.7
Yun et al. (2021)(9)	Retrospective cohort	Various	NA	57.51 (12.48)	816,811	NA	4.5	ICD-10 codes	NA	46.9	39.7	16.5	NA	NA	NA	3.1
Guha et al. (2022)(8)	Retrospective cohort	Breast	35.5% I 32.6% II 17.1% III 14.8% IV	78	85,423	Chemotherapy (anthracyclines, HER2-inhibitors, cyclophosphamide, taxanes, platinum-based agents), Surgery, Radiotherapy, Hormone Therapy	1	ICD-9-CM codes	NA	0	74.6	37.3	44.8	31.2	14.3	3.5
Beukema et al. (2022)(17)	Prospective cross-sectional	Oesophageal	NA	67.8 (NA)	40	Chemotherapy (CB, PTX), Radiotherapy, Surgery	7.3	NA	NA	NA	20	35	10	0	NA	30

NA = not available; SD = standard deviation; AF = atrial fibrillation; HTN = hypertension; DM = diabetes mellitus; IHD = ischaemic heart disease; CCF = congestive cardiac failure; 5-FU = 5-fluorouracil; CP = cisplatin; CB = carboplatin; PTX = paclitaxel; DXR = doxorubicin; ECG = electrocardiogram; ICD-10 = International Classification of Diseases, Tenth Revision; ICD-9-CM = International Classification of Diseases, Ninth Revision, Clinical Modification.

**Fig. 2:**
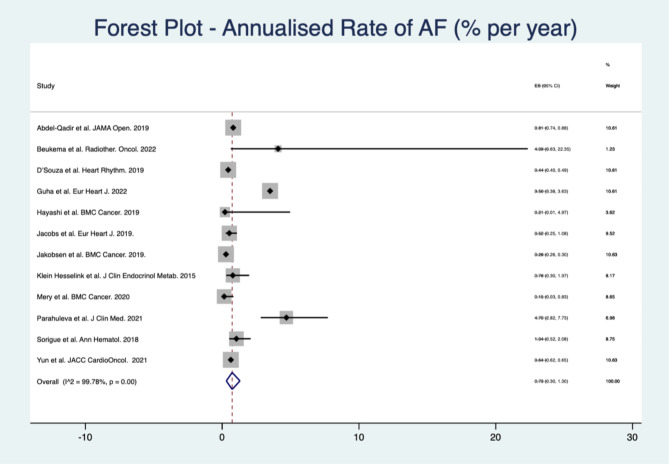
Forest plot showing the cumulative annualised risk of AF in cancer survivors

***Overall AF detection.*** The combined AF detection rate amongst all the studies was 4.7% (95% C.I 4.0-5.4%) (Fig. 2), which equated to a combined annualised AF rate of 0.7% (95% C.I 0.3–1.3%, n = 12 studies). There was significant heterogeneity between studies (*I*^*2*^ = 99.8%, p < 0.001).

In the breast cancer cohort (n = 6 studies), the combined annualised AF rate was 0.9% (95% C.I 0.1–2.3%), against with significant heterogeneity (*I*^*2*^ = 99.9%, p < 0.001). The overall certainty of evidence was low.

***Meta-regression.*** There was significant heterogeneity in the 16 studies, but we an exploratory meta-regression was performed (Table 2). Of the variables investigated, there were no significant associations noted with AF detection (p > 0.05).

**Table 2:** Features associated with the detection of atrial fibrillation

Variable	Number of studies	β (95% C.I)	P value
Age (years)	16	0.00 (-0.002– 0.002)	0.92
Hypertension (%)	13	-0.0001 (-0.0008–0.0006)	0.71
Diabetes Mellitus (%)	12	-0.0002 (-0.002–0.001)	0.81
Ischaemic Heart Disease (%)	7	-0.001 (-0.02–0.02)	0.88
Heart Failure (%)	7	-0.005 (-0.03–0.02)	0.66
Previous stroke (%)	3	-0.000001 (-0.00005–0.00005)	0.83
Male gender	14	0.00 (-0.00009–0.0002)	0.35
Study design	16	0.01 (-0.02–0.05)	0.43
Cancer type	16	0.0006 (-0.008–0.01)	0.89
Treatment type	11	0.04 (-0.15–0.24)	0.62

## Discussion

Our findings suggest an annual AF rate of ~ 0.7% in the overall cancer cohort after 12 months following treatment, with a is slightly higher incidence in the breast cancer cohort (0.9%). Overall, these findings suggest that AF risk in the cancer cohort are not significantly greater than in the general population. Our previous work demonstrated that single timepoint screening has an AF detection rate of around 1% [30].

A previous systematic review investigated the association between cancer and AF across multiple time points [31]. Whilst this review suggested an increased cumulative risk of AF in cancer patients, there were several limitations. We identified more relevant studies (n = 16 vs. n = 5) by using a more extensive search strategy which included searching more databases. The search terms used in the previously published systematic review only focussed on a single key term - “atrial fibrillation”, however we adopted a more sensitive approach by including more search terms encompassing all cardiovascular outcomes. Whilst the previously published systematic review included patients at different time points, we focused on cancer patients > 12 months post treatment to assess long term AF risk. The studies included in our systematic review are representative of patients seen in clinical practice, encompassing multi-ethnic cohorts (the included studies in the previously published review were all European patient cohorts). Interestingly, our findings are consistent with the subgroup analysis which demonstrated the risk of AF > 1 year post cancer diagnosis was not significantly elevated (OR = 0.97, 95% CI = 0.71–1.43) [31].

The main driver of increased AF risk was seen in the few months of cancer diagnosis [31]. There are several potential mechanisms. Cancer and the associated treatments (radiotherapy, chemotherapy, immunotherapy) may create a pro-inflammatory state with increased release of cytokines and chemokines. These inflammatory markers may play a role in accelerating cardiac structural remodelling, much faster than traditional AF risk factors such as diabetes or hypertension. Cancer treatments also have increased risk of electrolyte abnormalities and sepsis as well as the stressors associated with cancer surgery, which are known to increase AF risk. Some oncological treatments are associated with cardiotoxicity and may directly increase the risk of AF (e.g. ibrutinib) or indirectly via the development of heart failure (e.g. anthracyclines, anti-HER2 therapies and tyrosine kinase inhibitors). Patients having oncological treatment have more frequent follow-up and usually present multiple times to health care providers, providing more opportunities for diagnosis/screening, thereby creating a possible detection bias compared to the rest of the population [7]. There are also cancer specific factors which we do not fully understand with some cancer subtypes and those with advanced cancer stages having increased AF risk [8].

The overall AF detection rate in these studies was lower than what has been previously reported in unselected studies. AF risk and detection are highest in the first 90 days following diagnosis where patients are usually having cancer therapies which are associated with increased risk of cardiotoxicity and may have complications related to treatment [31]. Beyond the first year post cancer diagnosis, the risk of AF is known to be higher in specific cancer subtypes, most notably in haematologic malignancies and lung cancer, where it remains persistently elevated up to 5 years post-cancer diagnosis [9]. There are several possible explanations for the differences noted in our study. Our systematic review included patients > 1 year from cancer treatment, therefore the most unwell patients who die within 12 months of diagnosis would have been excluded. Patients who are > 1 year post-treatment may have fewer interactions with health career providers and may undergo less AF screening as a result, leading to lower detection rates. There is more active research being done in patients undergoing cancer treatment (who are within the first 90 days from clinical diagnosis), therefore cardiovascular screening and assessment is likely to be performed more frequently. There was significant heterogeneity between studies with significant differences in age, vascular co-morbidities and AF definitions. Despite the overall median age of patients in the studies was approximately 60 years, the presence of younger patients in some studies with low overall AF risk, may reduce the cumulative AF detection rate. Thus, while some subgroups may derive benefit, our results do not justify a generalized strategy of AF screening in all cancer patients.

AF screening has been recommended for patients ≥ 65 years on current European guidelines [11]. This recommendation is based on cohort studies demonstrating high detection rates of subclinical AF and cost-effectiveness with AF screening [32–35]. The development of newer screening technologies has been one of the biggest advances in AF screening. Most of the studies included in the systematic review used primarily AF diagnoses based on review of medical records or 12 lead ECG data from hospital admissions, thereby underestimating AF detection rates. Few studies used Holter monitoring for screening. The development of single lead ECG monitoring devices and smartwatches have created a paradigm shift in modern AF screening. We previously demonstrated that single lead ECG monitoring devices provided similar AF detection to Holter monitoring [30]. They also have the advantage of being cheap, easy to use and can provide screening over multiple time points, thereby increasing detection rates. These modern screening devices were not used in the majority of studies included in our systematic review, therefore AF rates in these cohorts may potentially be much higher.

Our study has important clinical implications. Our findings suggest that AF risk in patients in cancer remission may not be significantly elevated compared to the rest of the population. Whilst AF risk may be increased during cancer treatment and in patients with certain cancer types and those with advanced cancer stages, our results suggest that not all cancer patients have an elevated risk. Further prospective cohort studies are required to better delineate the group of patients at higher risk of developing AF so that screening programs can be more targeted thereby improving feasibility and cost-effectiveness. More frequent opportunistic AF screening in all cancer patients may not be required.

**Limitations.** There are several limitations. There was significant heterogeneity amongst the individual studies with regards to study design and patient demographics (although our meta-regression analysis did not find variables with a significant association with AF detection rate). Details of cancer treatments given were also poorly reported so it was unclear what specific treatments patients had received. Newer targeted therapies and immunotherapy agents are associated with multiple cardiovascular complications including AF so it is likely that the ongoing evolution in cancer treatment will lead to higher rates of AF in the future which is not accurately represented in our study.

AF definitions varied amongst studies and the lack of use of modern screening technologies such as smartwatches was a significant limitation. It is likely that most of the studies underestimated their AF rates as subclinical AF episodes were likely not captured due to lack of opportunistic screening. Medication history and risk factor management was not documented so it is possible that the use of cardioprotective medications such as beta blockers and ACE inhibitors may have reduced AF rates during follow-up. The risk profile of individual patients was difficult to ascertain as there was limited reporting of vascular risk factors, previous stroke and risk scores such as the CHA_2_DS_2_-VASC score were not routinely reported [10]. Some cancer types known to be associated with elevated AF risk such as lung cancer and haematological malignancies were not well represented in our systematic review.

**Conclusion.** Despite study heterogeneity, AF rates do not appear to be higher overall in cancer survivors compared to the general population. Further studies evaluating longer term incidence of AF in specific cancer subtypes is warranted.

## Electronic supplementary material

Below is the link to the electronic supplementary material.

Supplementary Material 1

Supplementary Material 2
