# Supplementary material for: Atrial fibrillation in cancer survivors – a systematic review and meta-analysis
Source: Cardiooncology. 2023 Jun 17;9:29. doi: 10.1186/s40959-023-00180-3 (PMC10276447; doi:10.1186/s40959-023-00180-3)
Supplement: Supplementary file 1 — Supplementary Material 1 [file 40959_2023_180_MOESM1_ESM.docx]

SUPPLEMENTAL MATERIAL

**Table S1:** PubMed search strategy

| # | searches |
| --- | --- |
| 1 | cancer*[Title/Abstract] OR tumor*[Title/Abstract] OR neoplasm*[Title/Abstract] OR malignan*[Title/Abstract] OR carcinoma*[Title/Abstract] OR neoplasm[MeSH Terms] |
| 2 | atrial fibrillation[Title/Abstract] OR ((atrial[Title/Abstract] OR atrium[Title/Abstract] OR auricular[Title/Abstract]) AND fibril*[Title/Abstract]) OR atrial fibrillation[MeSH Terms] OR cardiovascular events[Title/Abstract] OR cardiac outcomes[Title/Abstract] |
| 3 | 1 AND 2 |
| Filters: | English, Humans, Adults |

**Table S2:** Embase search strategy

| # | searches |
| --- | --- |
| 1 | cancer*.ab.kf.ti |
| 2 | tumor*.ab,kf,ti. |
| 3 | neoplasm*.ab,kf,ti. |
| 4 | malignan*.ab,kf,ti. |
| 5 | carcinoma*.ab,kf,ti. |
| 6 | 1 or 2 or 3 or 4 or 5 |
| 7 | atrial fibrillation.ab,kf,ti. |
| 8 | ((atrial or atrium or auricular) and fibril*).ab,kf,ti. |
| 9 | cardiovascular events.ab,kf,ti. |
| 10 | cardiac outcomes.ab,kf,ti. |
| 11 | 7 or 8 or 9 or 10 |
| 12 | 6 and 11 |
| 13 | 12 |
| 14 | limit 13 to (human and english language and (adult <18 to 64 years> or aged <65+ years>)) |

**Table S3:** Web of Science search strategy

| # | searches |
| --- | --- |
| 1 | TI=(cancer* or tumor* or neoplasm* or malignan* or carcinoma) AND TI=(atrial fibrillation or ((atrial or atrium or auricular) and fibril*) or "cardiovascular events" or "cardiac outcomes")) OR (AB=(cancer* or tumor* or neoplasm* or malignan* or carcinoma) |
| 2 | AB=(atrial fibrillation or ((atrial or atrium or auricular) and fibril*) or "cardiovascular events" or "cardiac outcomes")) OR (AK=(cancer* or tumor* or neoplasm* or malignan* or carcinoma) |
| 3 | AK=(atrial fibrillation or ((atrial or atrium or auricular) and fibril*) or "cardiovascular events" or "cardiac outcomes") |
| 4 | 1 AND 2 AND 3 |
| Filters | English |

**Table S4:** Study quality assessment using the Newcastle-Ottawa Scale (NOS).

| **Author/Year of publication** | **Selection** | **Comparability** | **Outcome** | **Final score** |
| --- | --- | --- | --- | --- |
| Rao (2012) | **** |  | ** | 6 |
| Hesselink (2015) | **** | * | *** | 8 |
| O’Neal (2015) | **** | ** | * | 7 |
| Chalazan (2016) | *** | ** | * | 6 |
| Sorigue (2018) | **** |  | ** | 6 |
| Abdel-Qadir (2019) | **** | ** | *** | 9 |
| D’Souza (2019) | **** | ** | *** | 9 |
| Jacobs (2019) | ** |  | *** | 5 |
| Hayashi (2019) | ** |  | *** | 5 |
| Jakobsen (2019) | **** | ** | ** | 8 |
| Mery (2020) | *** |  | ** | 5 |
| Li (2021) | **** | ** | * | 7 |
| Parahuleva (2021) | *** | ** | *** | 7 |
| Yun (2021) | **** | ** | *** | 9 |
| Guha (2022) | *** | ** | *** | 7 |
| Beukema (2022) | * |  | * | 2 |
